# Changes of urine metabolite profiles are induced by inactivated influenza vaccine inoculations in mice

**DOI:** 10.1038/s41598-019-52686-5

**Published:** 2019-11-07

**Authors:** Eita Sasaki, Hideki Kusunoki, Haruka Momose, Keiko Furuhata, Kazuo Hosoda, Kaori Wakamatsu, Takuo Mizukami, Isao Hamaguchi

**Affiliations:** 1 Department of Safety Research on Blood and Biological Products, National Institute of Infectious Diseases, 4-7-1 Gakuen, Musashi-Murayama, Tokyo 208-0011 Japan; 2 Department of Molecular Science, Graduate School of Science and Technology, Gunma University, 1-5-1 Tenjin-cho, Kiryu, Gunma 376-8515 Japan

**Keywords:** Inactivated vaccines, Preclinical research

## Abstract

The safety evaluation of vaccines is critical to avoid the development of side effects in humans. To increase the sensitivity of detection for toxicity tests, it is important to capture not only pathological changes but also physiological changes. ^1^H nuclear magnetic resonance (NMR) spectroscopy analysis of biofluids produces profiles that show characteristic responses to changes in physiological status. In this study, mouse urine metabolomics analysis with ^1^H NMR was performed using different influenza vaccines of varying toxicity to assess the usefulness of ^1^H NMR in evaluating vaccine toxicity. Two types of influenza vaccines were used as model vaccines: a toxicity reference vaccine (RE) and a hemagglutinin split vaccine. According to the blood biochemical analyses, the plasma alanine transaminase levels were increased in RE-treated mice. Changes in metabolite levels between mice administered different types of influenza vaccines were observed in the ^1^H NMR spectra of urine, and a tendency toward dosage-dependent responses for some spectra was observed. Hierarchical clustering analyses and principal component analyses showed that the changes in various urine metabolite levels allowed for the classification of different types of vaccines. Among them, two liver-derived metabolites were shown to largely contribute to the formation of the cluster. These results demonstrate the possibility that urine metabolomics analysis could provide information about vaccine-induced toxicity and physiological changes.

## Introduction

Vaccination is an important tool for preventing viral and bacterial infections. Vaccines can prevent infections by many viruses, including influenza virus, Japanese encephalitis virus and rabies virus. Thus, vaccines play an important role in protecting people from the threat of infectious diseases. Since vaccines are being administered to healthy people, the safety assessment of vaccines is important. Among the conventional safety tests for vaccines, the abnormal toxicity test (ATT), mouse body weight loss test, rabbit pyrogen test, and leukopenic toxicity test (LTT) have been conducted in Japan as animal safety tests^1^. The ATT assesses vaccine toxicity due to body weight loss and pathological changes. Leukopenic toxicity is observed when animals are injected with whole virion-inactivated influenza vaccine (WPV) and influenza viruses as a result of rapid interferon (IFN)α production within 24 h^2^. Thus, LTT has been employed for safety testing for inactivated influenza vaccines in Japan^1^. However, these methods mainly evaluate pathological and immunotoxicological changes. To increase the sensitivity of the detection of toxicity, it is important to capture not only pathological or phenotypic changes but also physiological changes. Thus, a new tool that can evaluate physiological changes is needed.

In recent years, as a alternative testing method to ATT, the development of a novel test method using the assessment of the gene expression profile in the lung has been carried out^3–7^. This method can be evaluated within a short time compared with that of the conventional ATT method, which requires 7 days for the test period, and can obtain highly accurate toxicological and immunological information compared with conventional ATT. Moreover, it has been shown that biomarker gene-based methods are applicable to various types of vaccines, such as adjuvanted and virosomal vaccines^5,8–11^. The tests that use these biomarkers can mainly evaluate innate immune responses and are expected to be applied to the evaluation of the immunogenicity of vaccines and adjuvants. In addition, the biomarker gene-based method has also been able to predict the leukopenic toxicity of influenza and adjuvanted vaccines^12^. Thus, the biomarker gene-based method has many benefits for toxicity and immunogenicity assessment; however, physiological information cannot be assessed with this method. In general, the assessment of physiological changes can determine toxicity with high sensitivity, and some physiological changes appear prior to pathological and other phenotypic changes^13,14^. Therefore, capturing physiological changes is important when performing toxicological assessments with high sensitivity^15–18^.

To detect physiological changes, safety evaluation methods using metabolomics technology have been developed for synthetic medicines and industrial chemicals^17–20^. In metabolomics analysis, the objective is to comprehensively analyze the small molecules (metabolites) contained in biofluids by nuclear magnetic resonance (NMR) analysis. Identified metabolites responsible for some physiological reactions can be used as indicators of toxicity^15^. In addition, metabolomics analyses provide information on injured organs and physiological activities based on information about the metabolite^15–17^. This approach also has an advantage because biofluid samples such as urine can be collected noninvasively. Therefore, it is easy to use for clinical application compared with gene expression analysis, which is required for biopsy samples. Because of this, metabolomics analysis can be considered useful for assessing toxicity in animals and humans.

Meanwhile, there have been no reports on the use of metabolomics analyses in vaccine safety evaluation. The aim of this study was to analyze urine metabolite changes by using a mouse body weight loss test and LTT with ^1^H NMR analysis for different types of influenza vaccines. The acquired metabolomics data were assessed to determine whether urine could be used to evaluate toxicity-related physiological changes.

In this study, the influenza hemagglutinin split vaccines HAV and WPV were used as model vaccines. Influenza vaccines can be roughly divided into 2 types of formulations: WPV and HAV^21^. Although WPV has a high capability to induce antigen-specific antibody production and cytotoxic T lymphocyte (CTL) activation, it may cause side effects such as fever and swelling in humans with increased frequency compared with HAV^22^ and is not currently used except for pandemic influenza vaccines. While the HAV formulation has been widely used for seasonal influenza vaccines because HAV causes side effects at a low frequency compared with WPV, its immunogenicity has been considered to be lower than that of WPV^22,23^. Because of this, WPV has been used as a toxicity reference vaccine (RE) for the LTT. Previous studies of biomarker development for influenza vaccine safety tests have also employed RE as a toxicity reference vaccine^3–5^.

Therefore, in this study, RE and HAV were administered by intraperitoneal injection into mice according to the methods used for the mouse body weight loss tests and LTT. The metabolites contained in urine were analyzed using ^1^H NMR spectroscopy. The acquired data were used for hierarchical clustering analysis and principal component analysis (PCA) to verify whether the physiological changes induced by the two different vaccine toxicities could be assessed by urine metabolite profiling. At the same time, blood biochemical data were obtained to compare the sensitivity of the metabolomics data and general toxicity markers. Based on these data, the merits of metabolomics analysis for vaccine safety testing were discussed.

## Results

### Leukocyte reduction levels and body weight reduction after influenza vaccination

**Figure 1:**
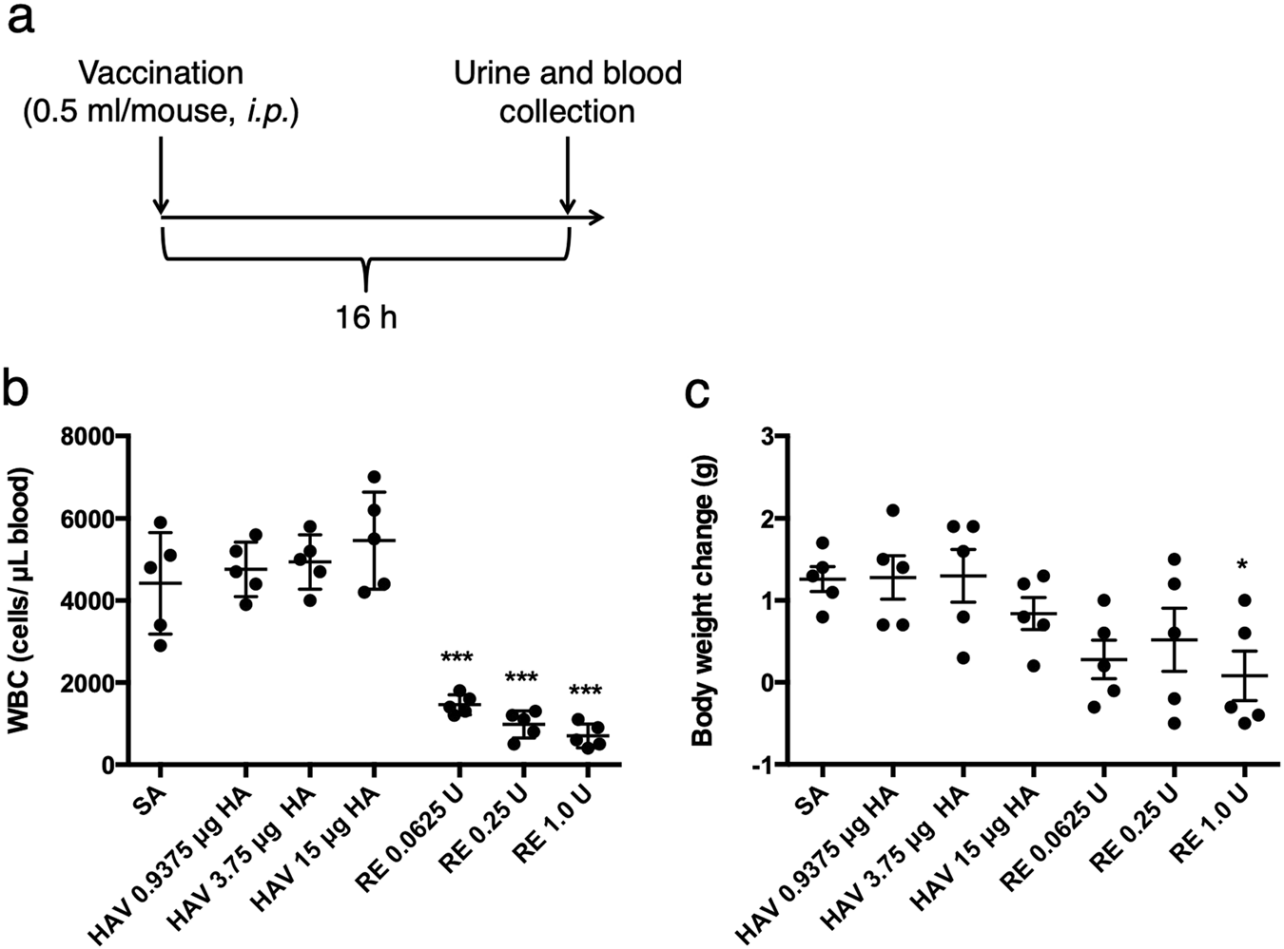
Leukopenic toxicity and body weight loss after influenza vaccine injection. Mice were intraperitoneally injected with 0.5 mL of each influenza vaccine. The concentrations of the toxicity reference vaccine (RE) were 0.0625, 0.25, or 1.0 U/mouse. The dose of hemagglutinin split vaccine (HAV) was 0.9375, 3.75, or 15 µg hemagglutinin (HA)/0.5 mL. Sterilized physical saline (SA) was used as the control. Sixteen hours after vaccination, blood was collected to assess the number of leukocytes. The animal experimental protocol used in this study is shown in (**a**). The body weight change was calculated by subtracting the weight before vaccination from the weight 16 h after vaccination. The data shown are (**b**) the number of leukocytes (indicated as white blood cells, [WBC]) and (**c**) the body weight changes. Each plot indicates the individual animals, and the error bar indicates the standard deviation (SD). **p < 0.01 and ***p < 0.001 (n = 5 in each group).

The experimental method was designed according to previous studies^4,5,8–10^ for the mouse body weight loss test and LTT. The reason for setting the analysis time point to 16 h was that peak WBC reduction has been observed 16 h after vaccination^24^. The maximum HA antigen content of HAV and RE was determined according to the LTT method^1^. Mice were intraperitoneally injected with 0.5 mL/mouse, and 16 h after vaccination, the body weight changes and the number of WBCs were measured (Fig. 1a).

The results showed that significant WBC reductions were observed at all doses in the RE-treated groups compared with the SA-treated group (Fig. 1b). Furthermore, body weight reductions were observed at all doses in RE-treated groups; however, significant changes were only observed at 1.0 U in the RE-treated group compared with the SA-treated group (Fig. 1c). While no dose in HAV-treated animals produced any significant changes in WBCs (Fig. 1b) and body weight compared with the SA group (Fig. 1c), at a dose of 15 μg HA, the HAV-treated group showed a tendency towards decreased body weight compared with the SA-treated group. These results indicate that 1.0 U of RE induced body weight loss and WBC reductions in mice 16 h after vaccination. Based on this result, urine metabolomics analysis was performed by using the abovementioned experimental design (Fig. 1a).

### Blood biochemical analyses after influenza vaccination

**Figure 2:**
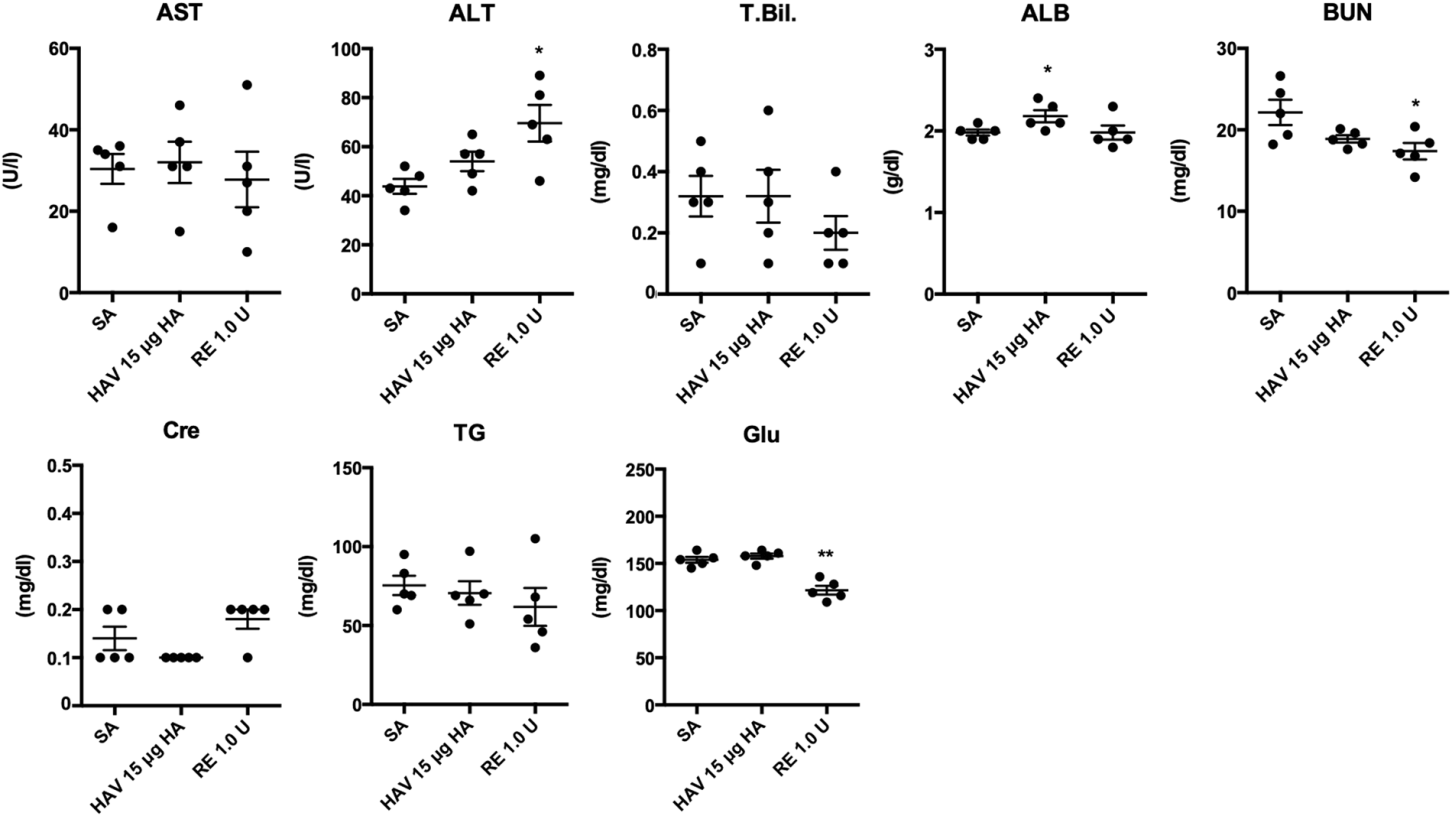
Blood biochemical changes after influenza vaccine injection. According to the method described in Fig. 1a, blood was collected from each mouse. The biochemical parameters (blood urea nitrogen [BUN], creatinine [Cre], alanine aminotransferase [ALT], aspartate aminotransferase [AST], total bilirubin [T. Bil], albumin [ALB], glucose [Glu], and triglyceride [TG]) in sera were measured by a biochemical analyzer. Each plot indicates the individual animals. *p < 0.05, **p < 0.01, and ***p < 0.001 (n = 5 in each group).

Blood biochemical data help with the evaluation of toxicological effects in animals. To assess the general toxicity of the influenza vaccine in mice, the serum levels of blood urea nitrogen (BUN), creatinine (Cre), alanine aminotransferase (ALT), aspartate aminotransferase (AST), total bilirubin (T. Bil), albumin (ALB), glucose (Glu), and triglyceride (TG) were measured in the SA-, highest dose HAV-, or RE-treated groups (Fig. 2). In these groups, a significant increase was observed in ALT, and a reduction was observed in BUN and Glu in mice treated with RA at a dosage of 1.0 U/mouse (Fig. 2). The HAV-treated group showed a significant increase in ALB levels (Fig. 2). These results suggest that a high dose of RE induced mild liver dysfunction in mice within 16 h because ALT is a cytoplasmic enzyme primarily found in hepatocytes, and the reduction in BUN levels suggests the failure of liver function^25^. A significant increase in serum ALB levels was observed in the HAV-treated group compared with that in the SA-treated group (Fig. 2). However, slight increases in serum ALB per se are insufficient to cause toxicological and physiological failure. These results indicate that treatment with 1.0 U/mouse of RE has mild physiological and cytotoxic effects on the liver; however, treatment with 15 μg HAV did not produce any changes in toxicity. To assess the strain-specific toxicity, we analyzed body weight changes, WBC changes and plasma ALT levels 16 h after vaccination with HAV or WPV comprised of type A influenza strains (H1N1 and H3N2) or type B influenza strains (B/yamagata) (Table 1 and Supplementary Fig. S1). For all strains, phenotypic changes were observed only for WPV vaccination but not for HAV vaccination. These results indicate that strain-specific toxicities did not produce phenotypic changes (Table 1). Based on this, it can be inferred that significant strain-specific changes in metabolites in urine do not occur. Therefore, the metabolomics analyses were performed using one strain of HAV.

**Table 1:** Body weight loss, leukopenic toxicity and plasma alanine aminotransferase (ALT) level changes after different strains of influenza vaccine injection.

	SA	HAVH1N1-15 μg HA	HAVH3N2-15 μg HA	HAV B/Yamagata-15 μg HA	WPV H1N1-15 μg HA	WPV H3N2-15 μg HA	WPV B/Yamagata-15 μg HA	RE 1.0 U
Body weight change (g)	0.98 ± 0.13	0.78 ± 0.25	0.53 ± 0.27	0.73 ± 0.18	−0.15 ± 0.13**	−0.05 ± 0.15**	0.05 ± 0.23*	−0.23 ± 0.15**
White blood cells (cells/μl blood)	5525 ± 464	5450 ± 312	6275 ± 415	6050 ± 184	1050 ± 64***	990 ± 112***	935 ± 129***	962 ± 170***
Alanine aminotransferase (U/l) activity	40.3 ± 6.4	43.8 ± 4	39.8 ± 6.9	41.8 ± 6.5	75.8 ± 13.5*	87.0 ± 9.2**	78.0 ± 12.1*	87.8 ± 8.7**

The unit of the dose for hemagglutinin (HA) in the hemagglutinin split vaccine (HAV) was μg and that for the toxicity reference vaccine (RE) was U.

Data are indicated as the mean ± SEM. (n = 4) *P < 0.05, **P < 0.01, and ***P < 0.001 compared with the sterilized physical saline (SA) group.

### Metabolomic analysis of mouse urine after influenza vaccination

**Figure 3:**
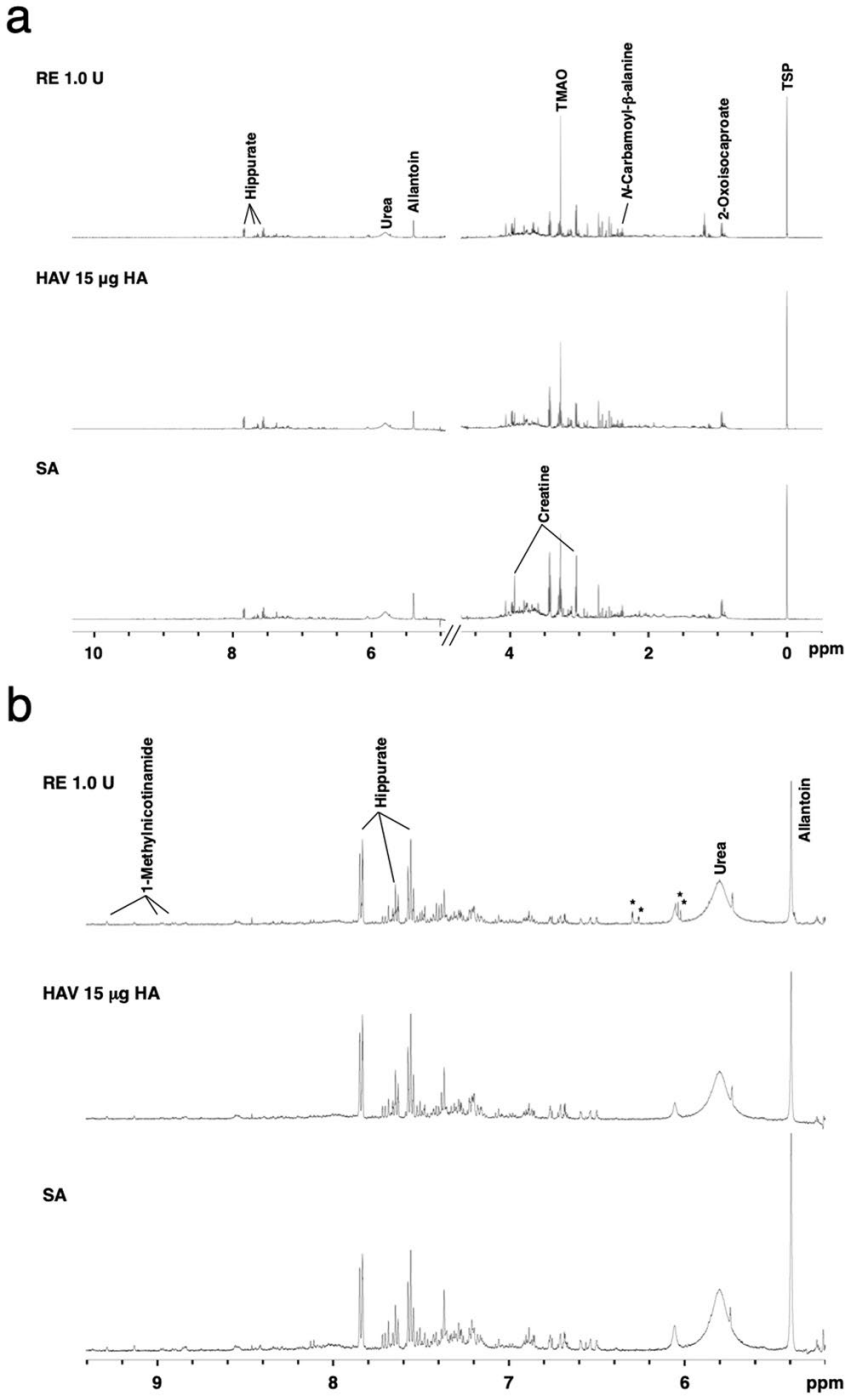
Five hundred megahertz ^1^H nuclear magnetic resonance (NMR) spectra of urine from mice treated with sterilized physical saline (SA), hemagglutinin split vaccine (HAV), toxicity reference vaccine (RE) in (**a**) the 0.0–10.5 ppm and (**b**) aromatic regions. *Asterisk indicates unidentified spectra. TMAO, trimethylamine *N*-oxide; TSP, trimethylsilyl propanoic acid.

Five hundred megahertz ^1^H NMR spectra of urine samples obtained 16 h after the vaccination of mice are shown in Fig. 3a,b. Visually, an increase in trimethylamine *N*-oxide was observed in urine from mice treated with 1.0 U of RE compared with SA-treated mice (Fig. 3a). In addition, four characteristic signals in the aromatic region were found only in the urine of RE-treated animals (Fig. 3b). However, these signals could not be assigned any metabolites during analysis using Chenomx NMR Suite software 8.4. We tried to use 2D NMR experiments to determine the urine metabolites. However, we could not obtain data for the determination of the metabolites. The possible reasons for this are that we did not have the appropriate cryoprobes and that the urine volume was limited. We think that further optimization is necessary before 2D NMR analyses using mouse urine. In this study, 32 metabolites were identified from the NMR signals from urine. In comparison to the SA group, significant changes were observed in 22 metabolites out of a total of 32 metabolites in mouse urine following treatment with each of the different concentrations of HAV or RE (Table 2). In the RE-treated group, the concentrations of hippurate, pyruvate, and trimethylamine *N*-oxide (TMAO) showed significant increases compared with those in the SA group. Among them, only the concentration of pyruvate showed a tendency towards an increased response to the dose of RE. The concentrations of tricarboxylic acid (TCA) cycle intermediates such as 2-oxoglutarate, citrate, and succinate showed slight increases compared with those in the SA group (Table 2). Among the metabolites related to glycolysis, the concentrations of pyruvate were significantly increased at all doses of RE and were not increased at any dose of HAV (Table 2). Conversely, the concentration of lactate was not changed in the RE group compared with that in the SA group, suggesting that in the RE-administered group, glycolysis was enhanced, and as a result, anaerobic respiration may not have been increased according to the changes in the lactate concentrations. In addition to endogenous metabolites, methanol and ethanol were also detected (Table 2). These metabolites are mainly derived from substances contained in the vaccine formulation.

**Table 2:** Urine metabolite levels in urine from mice treated with sterilized physical saline (SA), hemagglutinin split vaccine (HAV), toxicity reference vaccine (RE).

Concentration (μM) in urine							
Metabolite	SA	HAV 0.9375 μg	HAV 3.75 μg	HAV 15 μg	RE 0.0625 μg	RE 0.25 μg	RE 1.0 U
1-Methylnicotinamide	244 ± 28	252 ± 131	217 ± 79	117 ± 55**	185 ± 2*	253 ± 69	450 ± 126**
2-Oxoglutarate	1039 ± 365	1089 ± 441	1779 ± 824	996 ± 339	1443 ± 349	1156 ± 451	1378 ± 747
2-Oxoisocaproate	868 ± 159	901 ± 378	730 ± 164	485 ± 186*	684 ± 247	682 ± 149	856 ± 410
3-Indoxylsulfate	1654 ± 285	1449 ± 496	1218 ± 408	945 ± 243*	807 ± 369*	1264 ± 333	1702 ± 632
3-Methyl-2-oxovalerate	301 ± 82	339 ± 111	349 ± 100	214 ± 86	245 ± 92	313 ± 71	439 ± 207
4-Hydroxyphenylacetate	190 ± 47	195 ± 56	211 ± 113	92 ± 25*	156 ± 37	195 ± 37	331 ± 196
Acetamide	192 ± 15	263 ± 113	235 ± 97	146 ± 37*	173 ± 37	195 ± 51	343 ± 127*
Acetate	234 ± 91	288 ± 106	219 ± 134	181 ± 85	252 ± 109	242 ± 102	171 ± 74
Allantoin	9762 ± 1417	9478 ± 1903	8207 ± 2460	5173 ± 2036**	8202 ± 2079	8826 ± 2767	12951 ± 3787
Citrate	2714 ± 865	2971 ± 1262	4703 ± 2658	2445 ± 531	4608 ± 1204*	2722 ± 813	2606 ± 979
Creatine	2903 ± 637	1196 ± 535**	468 ± 339**	741 ± 444**	1703 ± 833	1398 ± 847*	2004 ± 669
Creatinine	1810 ± 209	1808 ± 838	1557 ± 840	1108 ± 302**	1423 ± 280	1573 ± 477	2151 ± 712
Dimethyl sulfone	290 ± 58	342 ± 130	299 ± 134	231 ± 84	229 ± 32	285 ± 74	313 ± 67
Dimethylamine	1478 ± 184	1180 ± 326	944 ± 516	791 ± 284**	1145 ± 125*	1089 ± 269*	1298 ± 382
Ethanol	270 ± 90	388 ± 61	485 ± 197	256 ± 121	220 ± 139	265 ± 209	996 ± 1347
Formate	154 ± 47	120 ± 28	92 ± 59	102 ± 33	177 ± 4	163 ± 95	139 ± 86
Guanidinoacetate	1180 ± 213	1128 ± 336	1011 ± 490	722 ± 206*	814 ± 276	898 ± 260	1337 ± 374
Hippurate	4507 ± 725	5026 ± 1946	5441 ± 2880	2982 ± 1084	2841 ± 321*	3635 ± 977	4658 ± 1133
Lactate	147 ± 18	169 ± 28	162 ± 48	88 ± 34*	126 ± 17	115 ± 28	123 ± 39
Methanol	143 ± 36	109 ± 24	100 ± 32	80 ± 31*	103 ± 7	101 ± 33	109 ± 44
Methylamine	378 ± 60	393 ± 160	365 ± 180	262 ± 81	244 ± 24*	313 ± 52	407 ± 87
N,N-Dimethylglycine	230 ± 32	150 ± 16**	71 ± 23***	138 ± 68*	150 ± 6**	144 ± 42**	108 ± 44**
N-Carbamoyl-β-alanine	2926 ± 434	2217 ± 127*	1675 ± 183**	1150 ± 516**	2688 ± 327	2770 ± 625	3253 ± 1150
N-Phenylacetylglycine	1046 ± 157	1095 ± 278	874 ± 160	474 ± 200**	552 ± 33**	779 ± 263	992 ± 309
Pyruvate	44 ± 7	49 ± 16	47 ± 11	38 ± 10	77 ± 13**	90 ± 17**	120 ± 44**
Succinate	193 ± 68	182 ± 53	226 ± 140	128 ± 53	218 ± 118	155 ± 66	258 ± 140
Taurine	14741 ± 1408	14314 ± 5113	11035 ± 4178	8415 ± 2559**	11660 ± 8045	9206 ± 3296**	11272 ± 7297
Trimethylamine	220 ± 191	201 ± 126	228 ± 199	137 ± 46	184 ± 44	324 ± 79	422 ± 148
Trimethylamine N-oxide	1815 ± 680	2139 ± 1205	1460 ± 780	1024 ± 414	1569 ± 297	1962 ± 467	3234 ± 775*
Urea	61965 ± 9321	52829 ± 9058	52021 ± 17790	45017 ± 14507	50041 ± 5589	54823 ± 9321	64359 ± 11147
Valine	37 ± 4	29 ± 7	26 ± 4*	29 ± 7	27 ± 3*	29 ± 6	42 ± 12
cis-Aconitate	632 ± 145	482 ± 213	949 ± 573	618 ± 243	666 ± 232	687 ± 197	1055 ± 328

The unit of the dose for hemagglutinin (HA) in the hemagglutinin split vaccine (HAV) was μg and that for the toxicity reference vaccine (RE) was U.μ.

Data are indicated as the mean ± SD. (n = 5 in the sterilized physical saline [SA] group; n = 4 [0.9375 μg HA], 3 [3.75 μg HA], or 3 [15 μg HA] in the HAV group; n = 3 [0.0625 U], 4 [0.25 U], or 5 [1.0 U] in the RE group). *P < 0.05, **P < 0.01, and ***P < 0.001 compared with the sterilized physical saline (SA) group.

### Hierarchical clustering analyses of urine metabolites

**Figure 4:**
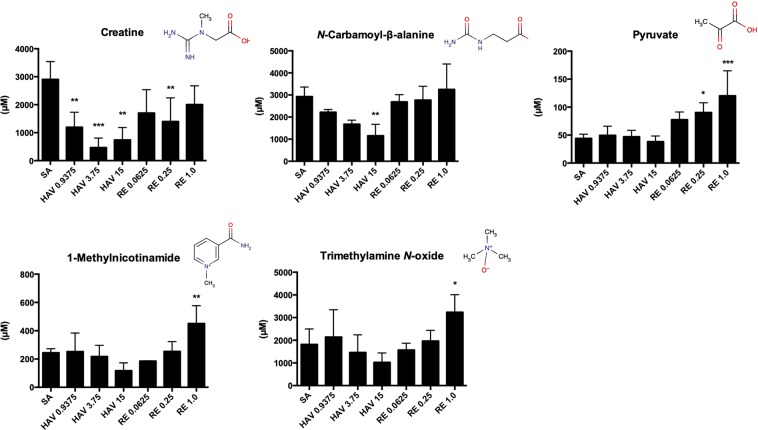
Changes in urine metabolite levels in urine caused by influenza vaccine injection. The metabolites were assigned and quantified according to the Chenomx NMR Suite 8.4 database as described in the Materials and Methods section. *p < 0.05, **p < 0.01, and ***p < 0.001 (n = 5 in the sterilized physical saline [SA] group; n = 4 [0.9375 μg HA], 3 [3.75 μg HA], or 3 [15 μg HA] in the hemagglutinin split vaccine [HAV] group; n = 3 [0.0625 U], 4 [0.25 U], or 5 [1.0 U] in the toxicity reference vaccine [RE] group).

Figure 4 shows typical metabolites that showed significant fluctuations depending on the vaccine that was administered. Each vaccine preparation showed characteristic fluctuations, and it was suggested that, except for *N*-carbamoyl-β-alanine and creatine, these metabolites could be used as indicators for the safety evaluation when RE is used as a toxicity control (Fig. 4).

**Figure 5:**
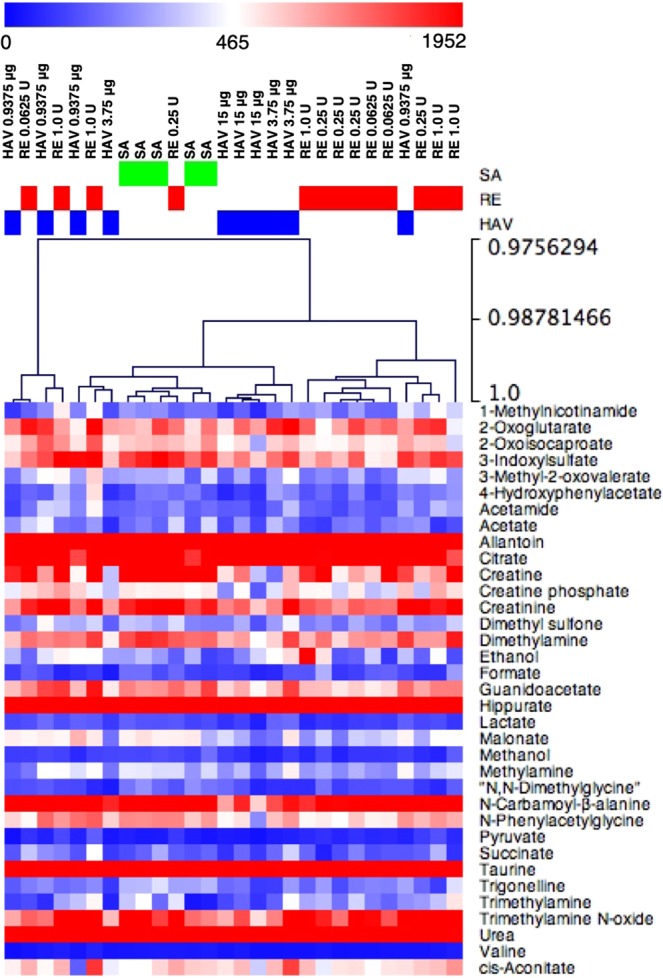
Hierarchical clustering analyses (HCA) of metabolomics data from mouse urine. Hierarchical cluster analysis of 32 metabolites in each animal treated with saline, hemagglutinin split vaccine (HAV), or the toxicity reference vaccine (RE). The log2 ratios and scale bars are shown in the resulting tree figure, which was obtained using the Multiple Experiment Viewer software. The values of the metabolite concentrations are listed in Table 2.

To comprehensively assess the changes in urinary metabolite concentrations caused by each vaccine formulation, hierarchical clustering analysis of the metabolites *vs* the vaccine types was performed. The changes in metabolites present in urine were broadly divided into RE and other clusters (Fig. 5). However, a complete match of the cluster classification according to the types of vaccine formulations could not be made according to the urine metabolite changes (Fig. 5). This was considered to be dependent on individual differences in the urine of animals. The result suggested that although the differences in urine metabolite profiles could be partially useful as an indicator of the safety of influenza vaccines, there are large individual differences in the levels of urine metabolites.

### Multivariate analysis of urine metabolites

**Figure 6:**
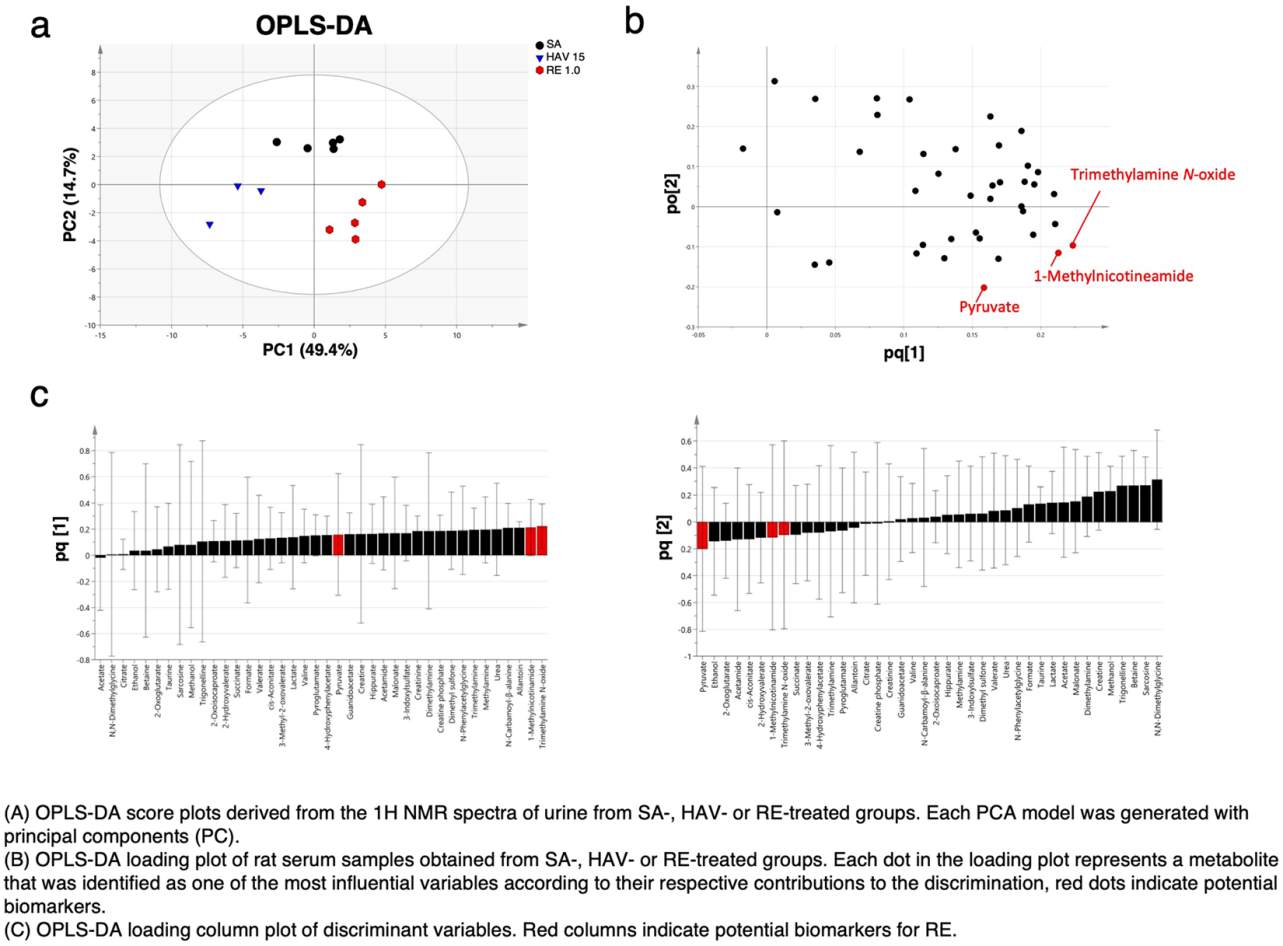
Principal component analysis (PCA) and orthogonal partial least-squares discriminant analysis (OPLS-DA) score plots derived from the metabolite concentrations in urine obtained from mice treated with sterilized physical saline (SA), 15 μg hemagglutinin (HA)-containing hemagglutinin split vaccine (HAV), or 1.0 U of the toxicity reference vaccine (RE). (**a**) OPLS-DA score plots derived from the ^1^H NMR spectra of urine from the SA-, HAV-, or RE-treated groups. Each PCA model was generated with principal components (PC), and each OPLS-DA model was generated with predictive components (T) and orthogonal components (TO) to discriminate between groups. (**b**) OPLS-DA loading plot of the mouse urine samples obtained from the SA-, HAV-, or RE-treated groups. Each dot in the loading plot represents a metabolite that was identified as one of the most influential variables according to its respective contributions to the discrimination of the groups; red dots indicate potential biomarkers. (**c**) OPLS-DA loading column plot of the discriminant variables. Red columns indicate potential biomarkers for RE.

To investigate whether the urinary metabolite profile differed depending on the type of influenza vaccine that was administered and which metabolites largely contributed to the differences in urine metabolite profiles, principal component analyses were performed. The urine metabolite profiles obtained from mice inoculated with SA and the highest doses of HAV or RE were analyzed with orthogonal projections to latent structures discriminant analysis (OPLS-DA) and classified according to vaccine type (Fig. 6a). The OPLS-DA model had an *R*^*2*^ value of 0.834 and a *Q*^*2*^ value of 0.608. The results showed that principal component (PC)1 generated a clear classification of HAV and RE. PC2 generated clear classification of SA and HAV or SA and RE. This indicates that there are different metabolites that have a large impact on the classification of SA and HAV as well as SA and RE. The loading plot showed that trimethylamine *N*-oxide and 1-methylnicotinamide, which were decreased by HAV and increased by RE (Fig. 4), had a large effect on PC1 (Fig. 6b). On the other hand, pyruvate, which was significantly elevated only by RE (Fig. 4), did not fluctuate in the presence of HAV; thus, its impact on the PC1 classification was smaller compared with that of trimethylamine *N*-oxide and 1-methylnicotinamide (Fig. 6c). The loading column plot also showed that changes in the urine trimethylamine *N*-oxide and 1-methylnicotinamide concentrations were better able to distinguish SA, HAV, and RE (Fig. 6c). To determine the influence of factors that fluctuated only for RE, profiling was performed by using PCA and OPLS analysis (Supplementary Fig. S2). As an OPLS model (*R*^*2*^ value of 0.943 and *Q*^*2*^ value of 0.641), classification tendency depending on the concentration of RE was observed by PC1 (Supplementary Fig. S2). Furthermore, it was shown that the factors having a large impact on the classification were trimethylamine *N*-oxide, 1-methylnicotinamide, and pyruvate (Supplementary Fig. S2).

According to the above data, the types of metabolites resulting from vaccination that fluctuated in urine differed between two types of formulations (HAV and RE). In addition, trimethylamine *N*-oxide, 1-methylnicotinamide, and pyruvate were identified as metabolites that reflected the properties of RE. To classify SA, HAV, and RE, trimethylamine *N*-oxide and 1-methylnicotinamide were considered as potential classification factors.

## Discussion

To date, the LTT, mouse body weight loss test, rabbit pyrogen test, and ATT have been used in Japan as safety tests for influenza vaccines^1^. However, conventional methods cannot assess the physiological effects of vaccines. In this study, we analyzed mouse urine to verify whether metabolomics analysis can be applied as a safety assessment tool for influenza vaccines. The reason for using urine is that it can be collected noninvasively; therefore, urine is also suitable for clinical applications^13^. Moreover, it is relatively easy to use urine for NMR analysis because it is less contaminated with proteins and lipids compared with serum and other biofluids^13^.

In this study, RE (comprised of WPV) and HAV were used as model vaccines. As reported, WPV induces leucopenia that peaks after 16 h and produces body weight loss after intraperitoneal injection into mice^24^. Additionally, in this study, RE vaccination produced marked leukocyte reduction and significant body weight loss compared with that observed in the SA group (Fig. 1b,c). Leukocyte reduction has been reported to occur in a manner dependent on IFNα production^2^. Thus, it has been thought that leukopenic toxicity caused by WPV occurs by an immunological mechanism^2^.

In the blood biochemical analyses, a slight increase in serum ALT levels, which is a marker of hepatocyte death, was observed (Fig. 2). Although it is difficult to determine whether fulminant liver injury is caused by RE administration on the basis of elevated levels of ALT (approximately average 60 U/l) (Fig. 2), it is presumed that mild damage or physiological stress was caused in the liver. In line with this result, autoimmune hepatitis caused by influenza vaccination in humans has been reported^25^. Furthermore, acute hepatitis and liver failure associated with influenza A infection in children has also been reported^26^. These reports suggest that influenza virus or its structural components (e.g., vaccine antigens) have the potential to affect liver functions or immune responses. However, the mechanism involved in hepatic dysfunction caused by influenza virus and its antigens has not been revealed. Further studies are needed to clarify the underlying mechanism. On the other hand, serum BUN levels were mainly used as a kidney injury biomarker. No significant increases in BUN were observed in mice administered 1.0 U of RE compared with that in mice in the SA group (Fig. 2). However, a decrease in BUN was observed in mice administered 1.0 U of RE (Fig. 2). Decreases in BUN are not notable changes in terms of kidney toxicity; however, a decrease in serum BUN levels is often observed in combination with low protein intake and decreased liver function^27^. Considering that ALT levels were increased by RE administration (Fig. 2), it is presumed that the decrease in BUN levels was caused by a decrease in liver function.

In addition to liver function, the serum glucose levels in the RE group showed a significantly decrease compared with those in the SA group (Fig. 2). However, the serum TG levels showed no marked change in the RE group (Fig. 2), suggesting that RE administration did not induce noticeable starvation. Because body weight loss in animals can be presumed to be caused directly by changes in food intake, it can be considered that the amount of activity in animals might be decreased because of an inflammatory reaction owing to leukopenic toxicity (Fig. 1b) and a slight increase in ALT levels (Fig. 2). However, in the HAV-administered group, there were no changes in blood biochemical markers suspected of being associated with toxicity (Fig. 2). Considering the above data, mild toxicity in the liver and changes in glucose metabolism caused by RE treatment were presumed to present according to the general toxicity markers.

The strain-specific toxicities of the influenza vaccines were examined. For the 3 different strains and 2 types of vaccine (HAV and WPV), strain-specific toxicities were not detected in terms of body weight changes, WBC changes and plasma ALT levels (Table 1 and Supplementary Fig. 1). Urine samples from vaccinated animals could not be analyzed in the present study because of the limited availability of samples for further analysis. However, there is a possibility that metabolomics analysis could detect toxicity that does not appear in the form of phenotypic changes (body weight changes, WBC changes and plasma ALT elevation); therefore, this should be investigated in future research.

**Figure 7:**
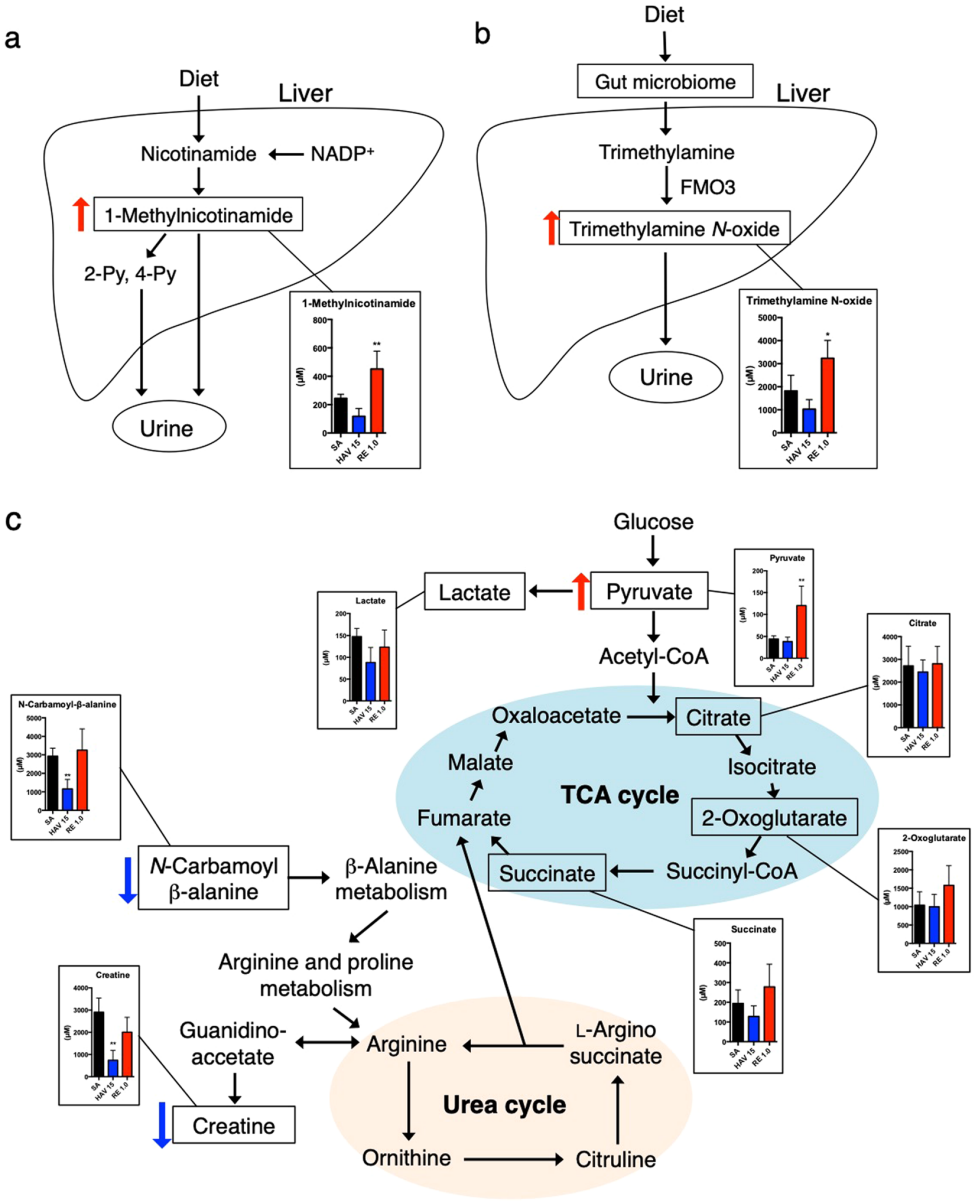
Representation of influenza vaccine-induced changes in urine metabolites related to nicotinamide (**a**) trimethylamine metabolism (**b**) and the tricarboxylic acid (TCA) cycle. (**c**) Each of the graphs shows a comparison of the urine metabolite levels from mice treated with sterilized physical saline (SA), 15 μg hemagglutinin (HA)-containing hemagglutinin split vaccine (HAV), or 1.0 U of the toxicity reference vaccine (RE). *p < 0.05 and **p < 0.01 (n = 5 in the SA or RE group and n = 3 in the HAV group).

Metabolites detected in urine have been known to be changed by damage to organs such as the liver and kidney and by cell metabolism^13–15^. Many studies have considered these changes as indicators of toxicity^16–19^. The liver is one of the major organs involved in endogenous metabolism (sugars, amino acids, and lipids) and the synthesis and excretion of endogenous metabolites. Metabolites excreted or released from hepatocytes are excreted in the urine through the portal vein^17^. The two metabolites that were increased in the RE administration group, 1-methylnicotinamide and trimethylamine *N*-oxide, are considered to be derived from the liver. Nicotinamide is known as niacin (vitamin B3) and is a component of nicotinamide adenine dinucleotide (NAD^+^) and its 2′-phosphate ester (NADP^+^), which is involved in the redox reaction in energy metabolism^28^. On the other hand, hepatocytes play a role in the metabolic excretion of nicotinamide. The methylation of nicotinamide is mainly performed in the liver^29,30^. Thus, 1-methylnicotinamide is produced in hepatocytes followed by its excretion in urine (Fig. 7a). Trimethylamine *N*-oxide is produced by the methylation of trimethylamine during the gut microbiome-mediated metabolism of amino acids^31^ (Fig. 7b). The metabolism of trimethylamine into trimethylamine *N*-oxide is mediated by flavin-containing monooxygenase (FMO) 3. Because FMO3 is strongly expressed in the liver^32^, trimethylamine *N*-oxide detected in urine would be mainly derived from the liver. The mechanism that causes these two metabolites to be increased in urine after RE vaccination is unclear. 1-Methylnicotinamide has been reported to be increased in urine from patients with liver cirrhosis compared with that in urine from healthy people^33^. It has been reported that the expression level of FMO3 in the liver is increased by liver injuries^34,35^. Because a slight increase in serum ALT levels was observed after RE administration (Fig. 2), it was also speculated that the increase in urinary trimethylamine *N*-oxide concentrations may have been due to the stressing of hepatocytes by RE administration. Collectively, it has been speculated that the elevation of 1-methylnicotinamide and trimethylamine *N*-oxide in urine may reflect slight liver damage caused by the administration of 1.0 U of RE. In terms of their usefulness as biomarkers for hepatic physiological changes, the sensitivity of these two metabolites is comparable to that of serum ALT levels (Figs 2 and 4). Therefore, urinary 1-methylnicotinamide and trimethylamine *N-*oxide levels cannot be used as highly sensitive biomarkers of hepatic physiological changes.

Urinary metabolites include intermediates in the TCA cycle and glycolytic systems. Among them, some metabolites showed changes that depended on the type of influenza vaccine administered (Fig. 7c). The urinary pyruvate concentration showed a significant increase after the administration of 1.0 U of RE. On the other hand, there was no change in the urinary lactate concentration in any of the groups. This suggests that although glycolysis still took place after vaccination with 1.0 U of RE, anaerobic metabolism did not occur. Pyruvate is synthesized from acetyl-CoA, which is an intermediate in the citric acid cycle. Three metabolites were detected that serve as intermediates in the citric acid cycle (Fig. 7c). Although no significant changes were observed in these 3 metabolites because of vaccination, a slight tendency towards increases in the 2-oxoglutarate and succinate concentrations was observed in the group administered 1.0 U of RE (Fig. 7c). Thus, it is suggested that during RE vaccination, glycolysis proceeds, and as a result, citric acid cycle metabolism may be slightly enhanced.

On the other hand, two urinary metabolites showed a significant reduction specifically in the 15 μg HAV vaccine-administered group: *N*-carbamoyl *β*-alanine and creatine (Fig. 4). *N*-carbamoyl *β*-alanine is metabolized by the urea cycle into arginine^36,37^. In the same manner as *N*-carbamoyl *β*-alanine, creatine is also metabolized into arginine by the urea cycle^38^. Considering that the urea cycle generates fumarate for the TCA cycle^35^, the decrease of both metabolites result in enhancing the TCA cycle. However, metabolites related to the TCA cycle were not increased in the HAV-treated group (Fig. 7c). Because of this, it is inferred that decreases in urine *N*-carbamoyl *β*-alanine and creatine in the HAV group did not directly reflect the enhancement of the TCA cycle. The reasons for these metabolite reductions by HAV vaccine administration could not be clarified by the metabolomics analysis conducted in this study.

By subjecting the detected metabolite concentrations to hierarchical clustering analysis, it was possible to classify the administered vaccines by profiling the metabolites in urine (Fig. 5). Two clusters, which were divided into the SA and HAV group and the RE group, were generated (Fig. 5). On the other hand, some clusters that did not conform to the vaccine types were also found, suggesting that individual differences among animals in terms of urine metabolites were influential in the hierarchical cluster analysis (HCA). This indicates that it was difficult to utilize urinary metabolites as highly sensitive indicators of the safety of influenza vaccines as an alternative to mouse body weight loss tests and LTTs.

This study is the first to report that changes in the profiles of metabolites in urine are caused by influenza vaccination and that liver-derived metabolites are responsible for leukopenic toxicity and body weight loss after RE administration. One new finding was that RE caused liver metabolic changes, suggesting that the liver is the principal organ impacted by the toxicological and physiological effects of influenza vaccines. This knowledge will aid in the development of alternative safety test methods for vaccines, as typified by the LTT and mouse body weight loss test, by utilizing the metabolomics analysis of *in vitro* cultured hepatocytes.

## Materials and Methods

### Reagents

Sterilized physiological saline (SA) was obtained from Otsuka Pharmaceutical Co. (Tokyo, Japan). Deuterium oxide (99.9% atom D) containing 3-(trimethylsilyl)-propionic-2,2,3,3-d4 acid and sodium salt (TSP, 0.05 w/v %) for NMR analysis was purchased from Sigma-Aldrich (St. Louis, MO, USA).

### Animals and ethics statement

Female 6- to 7-week-old BALB/c mice (16–22 g) were obtained from SLC (Shizuoka, Japan). All mice were housed in rooms maintained at 23 ± 1 °C with 50 ± 10% relative humidity and a 12-h light/dark cycle. The mice were acclimated for at least 3 days before use in experiments. All animal experiments were performed according to the guidelines of the Institutional Animal Care and Use Committee of the National Institute of Infectious Diseases, Tokyo, Japan. The study was approved by the Institutional Animal Care and Use Committee of the National Institute of Infectious Diseases.

### Influenza vaccines

RE is a toxicity reference reagent issued by the National Institute of Infectious Diseases (Japan). RE is a lyophilized whole-virion preparation of inactivated influenza virus, consisting of three different types of inactivated whole virions: A/New Caledonia/20/99 (H1N1), A/Hiroshima/52/2005 (H3N2), and B/Malaysia/2506/2004. RE is used as the toxicity reference for the LTT in Japan^1^. To generate the 1.0 U/0.5 mL RE solutions, freeze-dried RE was reconstituted in 12 mL of SA. For other concentrations of RE solution, an appropriate volume of SA was used. The HAV and WPV influenza A viruses (A/California/7/2009(X-179A)(H1N1)pdm09 or A/Switzerland/9715293/2013(NIB-88)(H3N2)) and influenza B virus (B/Phuket/3073/2013(B/Yamagata)) were kindly provided by Dr. Hideki Asanuma (Influenza Virus Research Center, National Institute of Infectious Diseases, Musashimurayama, Tokyo, Japan). HAV was reconstituted in an appropriate volume of SA and serially diluted with SA to prepare solutions of 0.9375–15 µg HA/0.5 mL. The HA antigen content was determined according to the LTT method in the Minimum Requirements for Biological Products Guidelines of Japan (JMR)^1^.

### Mouse body weight loss test and the LTT

The principal methods (Fig. 1a) used were the mouse body weight loss test and the LTT method as described in JMR^1^, with slight modifications. Briefly, mice were intraperitoneally injected with various concentrations of RE, HAV, or SA (0.5 mL/mouse). Then, 16 h after vaccination, the mice were anesthetized with pentobarbital followed by exsanguination through the inferior vena cava, and the sera were isolated using Capiject Capillary Blood Collection Tubes (Terumo Corp., Japan). The aliquot of blood was immediately transferred into an EDTA-coated tube (BD Vacutainer, Becton Dickinson, Franklin Lakes, NJ, USA) to measure the number of leukocytes. The number of leukocytes was counted with an automatic hemocytometer, the Celltac MEK-6450 (Nihon Kohden, Tokyo, Japan). All *in vivo* experiments were performed at the same time within a 2 h period to avoid fluctuations in urine components because of the light and dark cycle, which can affect feeding behavior in the breeding room.

### Urine collection

Kurien & Scofield^39^ described methods that involved the use of plastic wrap to collect pure urine from mice. According to their method, individual mice were placed onto clear plastic wrap and kept there until urination occurred. Approximately ≥100 μL of urine could be collected from 60–100% of the individuals in each group. The urine was immediately centrifuged at 2000 *g* for 10 min to remove gross debris and then stored at −80 °C until use for the NMR analyses.

### ^1^H NMR spectroscopic analysis

Urine samples were thawed at room temperature before use. After complete thawing and mixing, 50 μL of each urine sample was added to 450 μL of NMR solution containing 75 μL of 1.0 M potassium phosphate solution (pH 7.0) and 50 μL of D_2_O/TSP. The mixture was centrifuged at 7700 *g* for 3 min at 4 °C, and the supernatant (~500 μL) was transferred to a 5-mm NMR tube. All NMR experiments were performed on a Bruker AVANCE III 500 MHz spectrometer at 25 °C. All NMR spectra of the urine samples were acquired using a standard Bruker noesypr1d pulse sequence, and a total of 128 scans were collected to obtain 48,076 data points over a spectral width of 6009.615 Hz with a mixing time of 100 ms and an acquisition time of 4 s.

### Spectral data processing and multivariate statistical analysis

The metabolites in the spectra were assigned using Chenomx NMR Suite 8.4 software (Chenomx, Edmonton, AB, Canada). All NMR spectra were phased and baseline corrected, and the spectral binning data were generated using Chenomx NMR Suite 8.4 software. The region of the spectrum that included water (δ 4.6–5.0) was removed from the analysis for all groups to eliminate variation by increasing the water suppression efficiency. The quantification of the metabolites was performed using Chenomx NMR Suite 8.4 software. The resulting data sets were then imported into SIMCA-P 12.0 (Umetrics AB, Umeå, Sweden) for multivariate statistical analysis, orthogonal partial least-squares discriminant analysis (OPLS-DA), and PCA. PCA with an unsupervised pattern-recognition (PR) method was performed to examine the intrinsic variation in the data set, and OPLS-DA, a supervised PR method, was also employed to maximize the separation between each of the groups. The quality of the models was described by the *R*^*2*^ and *Q*^*2*^ values.

### Blood biochemical analyses

The serum BUN, Cre, ALT, AST, T. Bil., ALB, Glu, and TG levels were measured using DRI-CHEM 3030 (Fujifilm, Tokyo, Japan).

### Statistical analysis

For multiple comparisons, one-way analysis of variance followed by Dunnett’s multiple comparison test was performed. For the comparison of two groups, an unpaired Student’s *t*-test was performed. The statistical analyses were performed with GraphPad Prism 6 software (GraphPad Software, La Jolla, CA, USA). The hierarchical clustering analyses, including Pearson correlation and average linkage, were generated using the Multiple Experiment Viewer software package ver. 4.8.1 (http://mev.tm4.org).

## Supplementary information

Supplemental Figure 1 and 2

## Data Availability

The data generated during the present study are available from the corresponding author upon reasonable request.
