# Supplementary material for: Changes of urine metabolite profiles are induced by inactivated influenza vaccine inoculations in mice
Source: Sci Rep. 2019 Nov 7;9:16249. doi: 10.1038/s41598-019-52686-5 (PMC6838172; doi:10.1038/s41598-019-52686-5)

**Supplementary Information**

**Changes of urine metabolite profiles are induced by inactivated influenza vaccine inoculations in mice**

**Eita Sasaki, Hideki Kusunoki, Haruka Momose, Keiko Furuhata, Kazuo Hosoda, Kaori Wakamatsu, Takuo Mizukami, and Isao Hamaguchi.**

**Suppl Fig. S1.** (a) Body weight changes, (b) white blood cell (WBC) changes and (C) plasma alanine aminotransferase (ALT) levels 16 h after vaccination. Each plot indicates the individual animals. **p* < 0.05, ***p* < 0.01, and ****p* < 0.001 compared with the SA group (n = 4 in each group).


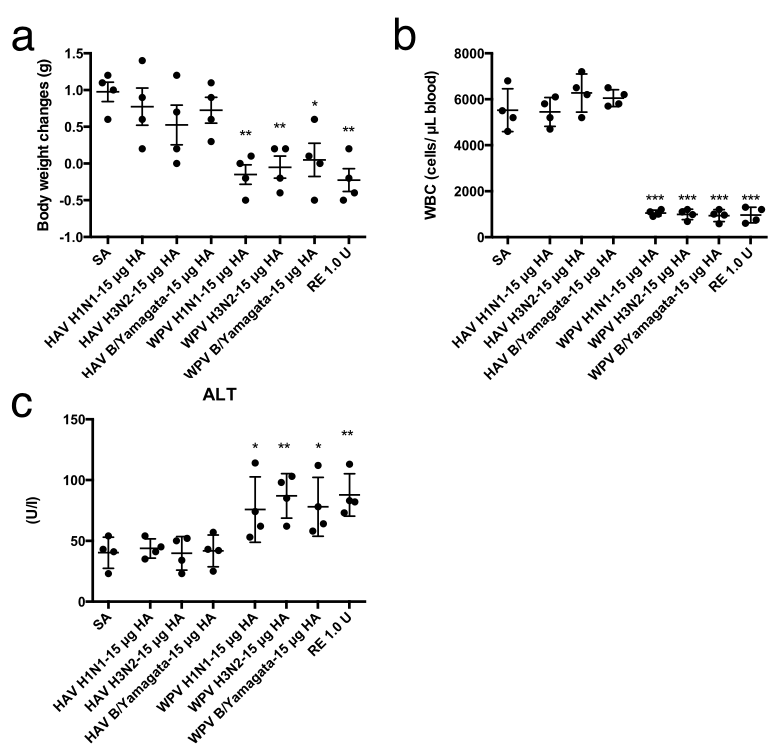


**Suppl Fig. S2.** (a) Principal component analysis (PCA) and (b) orthogonal partial least-squares (OPLS) score plots derived from the 1H NMR spectra of urine from the sterilized physical saline (SA)-, influenza hemagglutinin split vaccine (HAV)-, or toxicity reference vaccine (RE)-treated groups. Each PCA model was generated with principal components (PC), and each OPLS model was generated with predictive components (T) and orthogonal components (TO) to discriminate between the groups. (c) OPLS loading plot of rat serum samples obtained from the SA-, HAV-, or RE-treated groups. Each dot in the loading plot represents a metabolite that was identified as one of the most influential variables according to its respective contribution to the discrimination of the groups; red dots indicate potential biomarkers. (d) OPLS loading column plot of the discriminant variables. Red columns indicate potential biomarkers.


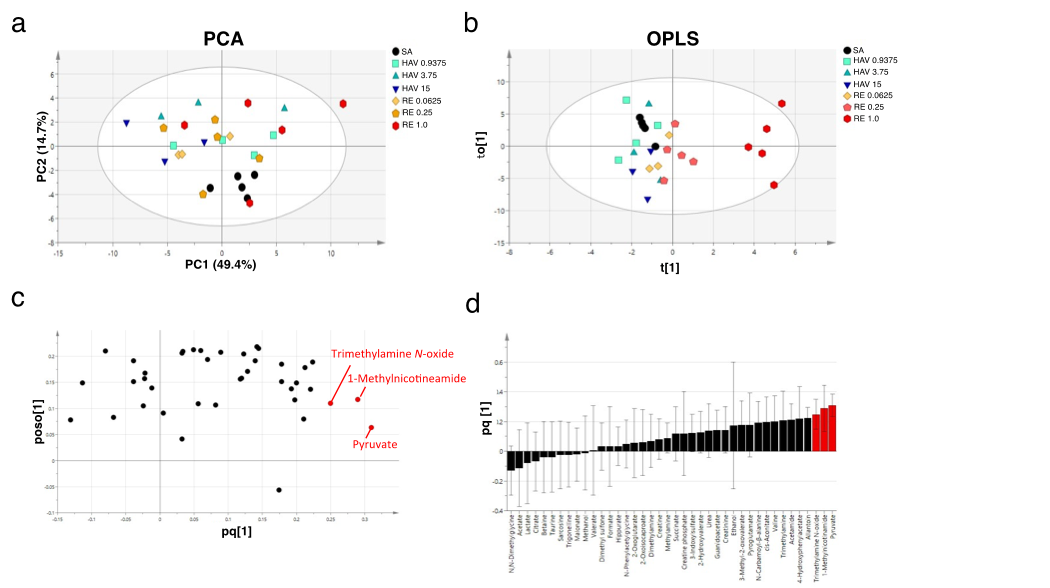

Supplement: Supplementary file 1 — Supplemental Figure 1 and 2 [file 41598_2019_52686_MOESM1_ESM.docx]
